# Members of Medical Societies No Longer Compelled to Take Association Journal

**Published:** 1910-05

**Authors:** 


### Members of Medical Societies No Longer Compelled to Take Association Journal.

According to a ruling of the Postoffice Department, Medical Societies can no longer compel members to subscribe for the society journal and the journal must have a paid subscription list or else be barred from second class postage rate. This is as it should be, for a great many physicians would have joined the A. M. A. but did not care for the journal, and could not afford to pay $5.00 for the membership alone, and they were inseparable, now the membership dues to the A. M. A. is $1.00 per year and subscription to the Journal is $4.00 per year. You now can join the A. M. A. and refuse the journal and vice-versa. The same rule applies to the State Association. The dues to the Oklahoma State Medical Association is $1.50 per year and the subscription to the Association Journal is 50 cents per year. It is not necessary to pay subscription to the journal to retain your membership in the Association so long as you pay your dues of $1.50 to the association.

This ruling of the Postoffice Department will make a big change in the A. M. A. and the journal. The membership of the A. M. A. will undoubtedly increase 25 per cent and the journal will lose at least 25 per cent of its present readers, thus making a 50 per cent difference in the membership and the subscribers to the Journal.—*Oklahoma Medical Journal.*

